# 15-Year Retrospective Study on the Success Rate of Maxillary Sinus Augmentation and Implants: Influence of Bone Substitute Type, Presurgical Bone Height, and Membrane Perforation during Sinus Lift

**DOI:** 10.1155/2023/9144661

**Published:** 2023-02-20

**Authors:** Vanessa Helena Jamcoski, Fernanda Faot, Raissa Micaella Marcello-Machado, Ana Claudia Moreira Melo, Flávia Noemy Gasparini Kiatake Fontão

**Affiliations:** ^1^Private Practice, Curitiba, Paraná, Brazil; ^2^Federal University of Pelotas, School of Dentistry, Department of Restorative Dentistry, Pelotas, Rio Grande do Sul, Brazil; ^3^Department of Prosthodontics and Periodontology, Piracicaba Dental School, State University of Campinas, Piracicaba, SP, Brazil; ^4^ILAPEO College, Department of Post-Graduation, Curitiba, Paraná, Brazil

## Abstract

**Objectives:**

To evaluate the success rate of bone grafts and implants carried out at the Latin American Institute for Research and Dental Education (ILAPEO), considering the following: (i) the different pure bone substitutes (autogenous, xenogeneic, and alloplastic), (ii) the presurgical bone height, and (iii) how the treatment is compromised when membrane perforation occurs during sinus lift in maxillary sinus surgeries. *Material and Methods*. The initial sample comprised 1040 records of maxillary sinus lifting surgeries. After evaluation, the final sample retained 472 grafts performed using the lateral window technique with a total of 757 implants. The grafts were divided into 3 groups: (i) autogenous bone (*n* = 197), (ii) xenogenous bovine bone (*n* = 182), and (iii) alloplastic material (*n* = 93). One calibrated examiner classified the sample into two groups based on the residual bone height (<4 mm and ≥4 mm) of the area of interest measured on parasagittal sections of tomographic images. Data on membrane perforation occurrences in each group were collected; qualitative variables were described using frequency, expressed as percentages. The Chi-square test was used to analyze the success of the graft types and the survival rate of the implants as a function of the grafted material and the residual bone height. The Kaplan-Meier survival analysis was used to calculate the survival rate of bone grafts and implants according to the classifications adopted in this retrospective study.

**Results:**

The success rate of grafts and implants was 98.3% and 97.2%, respectively. There was no statistically significant difference in the success rate among the different bone substitutes (*p* = 0.140). Only 8 grafts (1.7%) and 21 implants (2.8%) failed. There was a greater success rate for both grafts (96.5%) and implants (97.4%) when the bone height was ≥4 mm. The success rate in the 49 sinuses in which the membrane was perforated was 97.96% for the grafts and 96.2% for the implants. The follow-up periods after rehabilitation ranged from 3 months to 13 years.

**Conclusions:**

Within the limitations of the data analyzed in this retrospective study, maxillary sinus lift was a viable surgical technique that enabled implant placement with a predictable long-term success rate, regardless of the type of material used. The presence of membrane perforation did not interfere with the success rate obtained for grafts and implants.

## 1. Introduction

Extensive bone resorption and pneumatization of the maxillary sinus reduce the available implant options for rehabilitation of the posterior maxillary region [1, 2]. Bone grafting techniques that lift the maxillary sinus floor are still the most predictable and cost-effective technique to overcome these limitations [3]. The success rate of implants partially inserted into the grafted maxillary sinus is similar to those placed into native bone [4–6].

The success rate of implants installed directly in autogenous grafts and autogenous grafts mixed with other composites/synthetic bone grafts has been assessed in several systematic reviews in the last 10 years [7–15]. Overall, the results presented in these systematic reviews demonstrate that these implants have a high success rate, irrespective of the graft material used [16]. Moreover, no differences according to patient age, sex, healing time, and type of membrane or surgical graft were identified. Most systematic reviews [4–16] that compared different surgical techniques or the use of different surgical tools to perform maxillary sinus floor augmentation did not report significant differences regarding the implant insertion time, the use of bone grafts, the type of bone grafting material, and the mean bone height. One exception was the treatment ranking based on the evidence network by Papageorgiou et al. [17], which indicated that autografts presented the highest percentage of new bone, followed by synthetic grafts, xenografts, and allografts.

The different types of grafting each have their intrinsic advantages and disadvantages. Although autogenous bone is considered a surrogate material capable of promoting the activation and release of growth factors [18], when it is used in maxillary sinus augmentation surgeries, up to 45% of the graft reabsorbs around the implants during the first 24 months after the graft [19, 20]. In contrast, reabsorption of organic or synthetic bone grafting materials is comparatively lower [21–23]: approximately 18% to 22% in the first 24 months [24] and 10 to 20% after two years of loading [20]. Reducing the volume of grafts with autogenous bone does not seem to compromise the placement or success of implants [19, 20]. Alloplastic, homogenous, and xenogenous bone substitutes cause less morbidity to the patient [25] and can be safely used [16, 26, 27] when they are immunologically inactive, physiologically stable, and biocompatible, enabling osteogenesis and osteoconductivity [28]. Although beta-tricalcium phosphate (*β*-TCP) is an acceptable bone substitute to augment the maxillary sinus [29], the bone formation rate can be slower than in autogenous bone due to its low osteoconductivity [25, 30]. Lastly, bovine xenogenous bone diffuses gradually with newly formed bone at the interface during the biomaterial reabsorption process and is biocompatible and osteoconductive when used as a bone substitute for maxillary sinus lift [27].

In addition, the patient's bone characteristics can also influence the treatment options and outcomes. When the bone height is at least 5 mm, implant placement can be carried out in a single surgical procedure (maxillary sinus augmentation and simultaneous implant placement). However, when bone height is between 1 and 4 mm, a two-phase surgical procedure consisting of maxillary sinus augmentation and subsequent implant placement is recommended [31]. Implant failure rate is higher when there is a single surgical procedure after a maxillary sinus graft with residual bone between 1 and 3 mm [32, 33]. A clinical study by Lo Giudice et al. [34] evaluated the success rate of 45 dental implants placed on grafted bone with a residual bone height of 2 mm using two-phase surgical procedures for 5 years. Six months after the installation of the prosthetic crowns, the success rate of the inserted implants was 99.5%, suggesting that maxillary sinus surgery with bone grafting achieves excellent results, even when there is an atrophic bone crest [34].

Perforation of the sinus membrane is the most common surgical accident during grafting of the maxillary sinus, with reported incidence rates mostly between 20% and 25% [35]. Small perforations are easier to correct without compromising the success of the bone graft or the implants [36, 37]. Knowledge of the exact size of the sinus membrane perforation is essential for deciding on the right treatment plan [38] to increase the success rate: when the size is less than 5 mm, they should be covered with collagen membrane; when the size is larger than 5 mm, they can be sutured [39] to correct the perforation and to contain the bone graft and avoid reduction in bone volume [40]. Nonetheless, this complication can extend the duration of surgery and increase incidence of postoperative complications [41] and the risk of implant failure [42]. However, recent systematic reviews concluded that neither the Schneiderian membrane perforation during maxillary sinus floor augmentation procedures with lateral approach nor the presence of mucosal thickening pose a risk factor for sinus augmentation and implant survival rate [38, 43].

The main challenge in the grafted posterior areas of the maxilla is to keep the postsurgical bone resorption to a minimum after implants installation [44]. Higher marginal bone loss has been described in these areas compared with implants placed in native bone, especially during the first 12 months after functional loading [44]. Because implants must survive for extended periods and the resulting bone loss may affect the success rate, it is necessary to monitor the bone stability around the apical portion and the marginal area of the implant where the sinus membrane lifting procedure with grafting is performed, especially for long-standing loaded implants for which a reduced osseointegration could jeopardize survival [45].

In summary, more data are still needed to better understand the long-term influence of bone substitute type, presurgical bone height, and the membrane perforation during maxillary sinus lift on the treatment outcome of implants and bone grafts. Therefore, this retrospective study aimed to evaluate the medium- to long-term success rate of bone grafts and implants carried out at the Latin American Institute for Research and Dental Education (ILAPEO), considering the following: (i) the type of pure bone substitute (autogenous, xenogeneic, or alloplastic), (ii) the presurgical bone height, and (iii) the membrane perforation occurrence during sinus lift in maxillary sinus surgeries.

## 2. Material and Methods

This retrospective cohort study evaluated the success rate of autogenous, xenogenous, and alloplastic materials used as bone substitutes in maxillary sinus surgeries with simultaneous or nonsimultaneous placement of dental implants determined by the amount of residual bone based on respecting the 4 mm of residual bone remnant to adopt the one-stage surgery and the influence of residual bone height and sinus membrane perforation on sinus graft and implant success rates. Participants of the study had sought treatment at the ILAPEO (Latin American Institute for Research and Teaching in Dentistry), Curitiba/PR-Brazil, between July 2002 and November 2017, and the project was written as recommended by the STROBE (Strengthening the Reporting of Observational Studies in Epidemiology) guidelines [46]. The Research Ethics Committee of the Institute of Neurology of Curitiba (INC) approved this study (Protocol number 1230428).

### 2.1. Brief Description of the Surgical Procedures

This study employed the lateral window technique during surgery. The surgeries were performed under aseptic conditions using a regional blockade of the posterior superior alveolar, infraorbital, and greater palatine nerves and a terminal infiltrative at the bottom of the vestibule and palate to anesthetize. When the maxillary sinus floor did not meet the ridge crest or when there was a minimal area of keratinized tissue, the lateral wall of the maxillary sinus was accessed through a full thickness mucoperiosteal flap incised over the alveolar crest or the slightly palatalized ridge crest. The length and divergence of the anterior and posterior relaxing incisions were chosen to provide access to the region of interest while allowing a good baseline blood supply to the flap. To avoid flap dehiscence, the incisions were located at some distance from the proposed antrostomy, which was subsequently planned based on clinical evaluation and tomographic images.

The Schneiderian membrane was then accessed through the delimitation of the osteotomy that was performed with a minimal exposure of the sinus membrane to facilitate its elevation with adequate visibility, at 3 mm from the anterior wall and floor of the maxillary sinus, considering that the main blood supply for the future graft stems from the bone walls and not from the Schneiderian membrane. The osteotomy was performed using a straight hand piece with a 2 mm spherical diamond drill at 22000 rpm. Drilling was performed with anterior-superior and lateral-lateral movements, without applying pressure on the bone wall to avoid perforating the sinus membrane, until a slight movement of the “bone island” was perceived and a dark shadowed area became visible. From this moment on, detachment and removal of the bone window and adequate elevation of the Schneiderian membrane were performed.

With the exposure of the receptor site, bone substitutes from the 3 groups (autogenous, xenogenous, and alloplastic) and respective brands were emplaced to fill the maxillary floor. The flaps were repositioned, starting with the suturing of the relaxing incisions and ending with the suturing of the incision over the alveolar crest, with synthetic and absorbable Vicryl (polyglactin 910) 4-0 suture (Johnson & Johnson). After the grafting stage, the patients were maintained under postoperative control. The treatment of sinus membrane perforations was performed using a collagen membrane (Colla Tape, Zimmer) to cover the perforated region. The membrane was sutured respecting a safety margin of 1-2 mm, followed by an 8 month healing period to allow the sinus membrane to repair and repeat the procedure.

For the second-stage surgeries, CBCTs were performed to verify the bone dimensions for implant installation with a total flap incision. Implant diameters were selected based on the local bone availability and the desired insertion torque of at least 45 Ncm, as determined by a torque ratchet (Manual Torque Ratchet, Neodent). All implants were installed according to the manufacturer's recommended standards and guidelines, avoiding excess torque. Complications that occurred during execution of the procedures, such as membrane perforation, exaggerated graft resorption, and partial or total loss, were documented as an integrant part of the analysis of the results of these reconstructive procedures, along with installation of prosthetic crowns and follow-ups during periodic returns.

### 2.2. Data Collection

The initial sample consisted of 1040 records of maxillary sinus surgeries. During the data selection, the following inclusion and exclusion criterion were applied:

- Inclusion criteria: (1) patients with CBCT (cone bean computed tomography) in the region of interest prior to maxillary sinus lift surgery, as this enables to evaluate the residual bone height; (2) medical forms enabled collection of the following data: type of bone grafts, height of residual bone, surgical procedure, complications during the surgical procedure, and number of implants installed
- Exclusion criteria: (1) patients with medical records without the necessary information to perform the research (patients with incomplete list of drugs for systemic use to treat listed conditions, patients under medical treatment at the time of surgery, and patients with a factor that precludes or contra-indicates oral surgery); (2) grafts with mixed bone substitutes. In accordance with the rules of the ILAPEO institution, periodontal diseases were not considered a basis for exclusion, conditional on periodontal treatment prior to surgery and adherence to treatment until the end of this research

After this screening, the final sample consisted of 382 patients with 472 bone grafts. Of these 472 grafts, 197 were autogenous bone grafts (group 1), 182 were xenogenous bovine bone grafts (group 2), and 93 were alloplastic material (group 3) from different manufacturers (Table 1). The treatment of perforations was performed using a collagen membrane (Colla Tape, Zimmer) to cover the perforated region. The membrane was then sutured respecting a safety margin of 1-2 mm, followed by an 8 month healing period to allow the sinus membrane to repair and repeat the procedure.

Between 2002 and 2017, a total of 757 implants were installed onto the grafts, the distribution of implants was always chosen closer to the position in the arch where there previously were natural dental elements, and the width of the implant respected the mesiodistal distance between implants or between teeth and implants. A total of 171 implants (22.6%) were rehabilitated with single prosthetic crowns, and 586 implants were rehabilitated with (77.4%) full arch or partial prostheses produced with metal-ceramic systems. The follow-up time varied between 3 months and 13 years.

The characteristics of the participants of this study are described below according to the treatment steps:

- Bone graft and implants in one surgical procedure: after the evaluation of the case, the maxillary sinus was augmented using one of the three bone substitutes, and implants (Neodent, Curitiba-PR, Brazil) were installed in the same surgery
- Bone graft and implants in two surgical procedures: a second CBCT was carried out to evaluate if the height of the graft enabled the placement of implants, which was carried out on average after ±9.6 months
- Second stage surgery and prosthetic installation: after an average period of 13.6 months, surgeries were carried out to expose the implant to the oral environment for prosthesis installation. The reopening time of the implants and installation of the prostheses varied according to the initiation of new postgraduation courses during which the procedures were carried out
- Follow-up consultation: all patients were requested to return to the institution for annual follow-up after loading of implants with prostheses, but the time between each appointment was individualized and dependent on the patient's ability to maintain adequate biofilm control. During the consultation, the prosthesis was removed for the prophylaxis and clinical examination, and the following aspects were evaluated: mobility, suppuration, pain, or any other complaint reported by the patient. Panoramic and periapical radiographs were taken to assess peri-implant bone conditions and potential loss of the graft or implant

#### 2.2.1. Criteria for Evaluating the Success of Grafts and Implants

The study sample was divided into three groups according to the origin of the bone substitute used: group 1 (autogenous bone), group 2 (xenogenous bone), and group 3 (alloplastic bone). Within the same sample, perforated sinuses were recorded separately to observe if there was a relationship with loss of graft and implants and the influence of the residual bone height.

Grafts were considered successful when they enabled the installation of the implants; implants were considered successful when they did not present pain, mobility, or suppuration during the follow-up period [33, 34] and allowed prosthetic rehabilitation and masticatory function. Conversely, grafts were considered unsuccessful when there was infection or reabsorption of the grafted material in the maxillary sinus, and implants were considered unsuccessful if there was infection and lack of osseointegration [33, 34].

#### 2.2.2. Data Sources and Measurements: Medical Records and CBCT

The dental forms were evaluated to obtain clinical information about the patient, graft, and status of implants such as graft material type, membrane perforation during surgery, pain, suppuration, and implant mobility.

**Figure 1:**
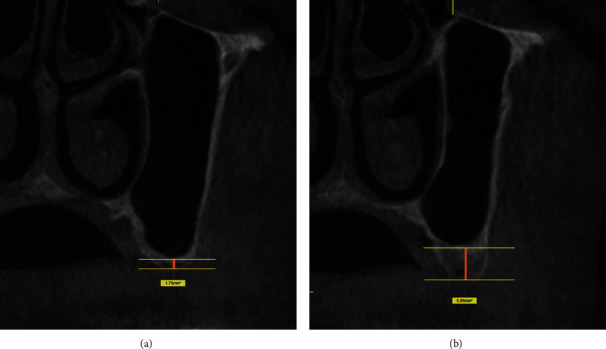
Representative examples showing the measurement of residual bone height in the tomographic image: (a) <4 mm and (b) ≥4 mm.

One calibrated examiner classified the residual bone into two groups (<4 mm and ≥4 mm) by measuring the area of interest on the parasagittal sections of the tomographic images (Galileos and Orthophos, Sirona, Bensheim, Germany), using the Sidexis XG and Galaxis software (Sirona). A reference line was drawn at the midpoint of the greatest alveolar ridge height, parallel to the floor of the maxillary sinus, and another line on the surface of the ridge. The alveolar ridge height was measured by joining the reference lines (Figure 1). One month after the first collection, CBCTs of 30 patients were randomly selected, and the same examiner made new measurements of the bone residual height to check the operator error. The systematic methodological error was evaluated using Student's *t*-test for paired samples and the random error was estimated using the Dahlberg formula with error estimated at 0.24.

#### 2.2.3. Statistical Analysis

The results of quantitative variables were described via the mean, median, minimum value, maximum value, and standard deviation. Qualitative variables were described using frequency, expressed as percentage. The Chi-square test was used to analyze the survival rate of the implants as a function of the grafted material and the height of the residual bone. The normality of the data distribution was assessed using the Kolmogorov-Smirnov test. *p* < 0.05 was considered statistically significant for all tests. The data were analyzed using the IBM SPSS Statistics v.20 software.

The Kaplan-Meier survival analysis [47] was used to calculate the survival rate of bone grafts and implants for each group. The survival analysis is the study of an observed item (bone grafts or dental implants) when a well-defined event (failure) occurs after some time. This analysis is ideal for longitudinal binary survival studies and is characterized by different follow-up times between individuals (placement of bone graft and/or dental implants) and loss of patient follow-up [48]. All bone grafts and implants that were not lost until the end of the observation period were assessed. The statistical comparison of survival curves for all groups was achieved by robust log-rank testing, which accounts for the cluster effects of multiple bone grafts and implants placed in a single patient [49, 50]. Statistical significance was considered at the 0.05 level.

## 3. Results

The mean age of the 382 patients was 50.8 years, and 73.3% were females. Of the final sample of 472 sinuses, 49 presented membrane perforation suitable for grafting. Of the 757 implants installed, only 373 were rehabilitated with prostheses; the remaining 384 implants were not rehabilitated because the patients did not return to the institution.

**Figure 2:**
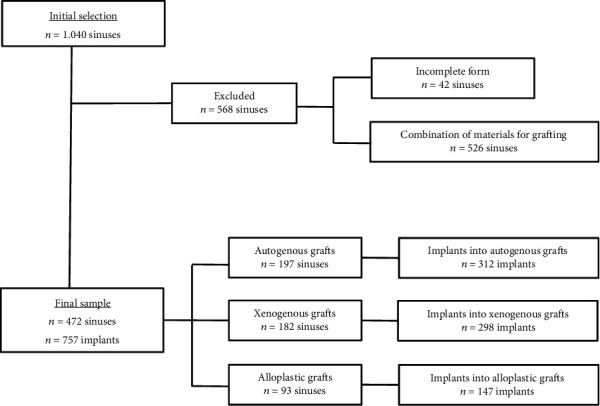
Flowchart summarizing the sample selection methodology applied in this retrospective study.

The selection of the study sample is summarized in a flowchart (Figure 2). The grafts used autogenous bone (G1 = 197), bovine xenogenous bone (G2 = 182), and alloplastic material (G3 = 93) from nine commercial brands. A total of eight (1.7%) grafts were lost: seven due to infection and one because of insufficient height to allow implant installation. Of the 757 implants, 21 (2.8%) were lost: 14 during the early period of osteointegration due to infection with clinical manifestation of suppuration and 7 during the late period of osseointegration with no other clinical manifestation as mobility during the implant loading phase. The implants in autogenous bone grafts presented the highest loss rate (3.53%; Table 2). There was no statistically significant association between the loss of implants and grafts and the origin of the grafted material.

A total of 255 grafts were carried out in residual bone smaller than 4 mm and 217 grafts in residual bone ≥ 4 mm. The residual bone height range in patients with residual bone heights ≥ 4 mm was 4-11 mm, while in patients with residual bone heights < 4 mm was 0.63-3.88 mm. Data on the success rate between grafted material and residual height on graft and implants are listed in Table 2. In all 49 sinuses, small membrane perforations (<10 mm) occurred during surgery. This complication was successfully treated, allowing subsequent grafting. Only one bone graft one was lost in the autogenous bone group. In these grafted maxillary sinuses, 79 implants were installed, and three implants failed. The success rate of grafts and implants according to the occurrence of membrane perforation is also presented in Table 2. The duration of follow-up consultations was as follows: 191 implants were followed up to one year, 97 up to five years, 59 up to eight years, and 31 continued to be followed up eight years after the grafting, totaling 378 implants with prosthetic rehabilitation (Table 3). According to the type of rehabilitation, no implant losses occurred.

**Figure 3:**
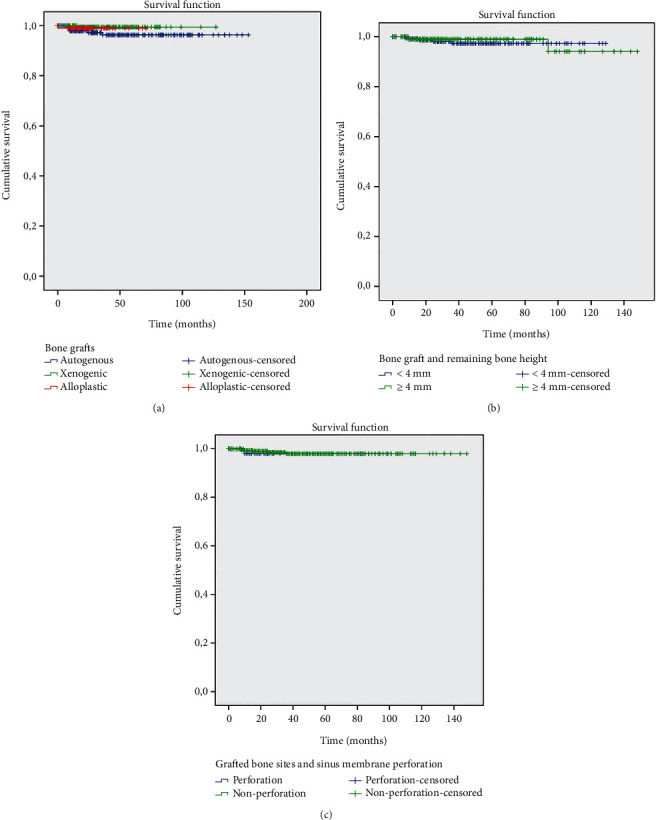
The Kaplan-Meier survival analysis. (a) Survival rate according to bone graft materials over time. (b) Relation between bone graft materials and remaining bone height: <4 mm and ≥4 mm. (c) Relation between grafted bone sites and sinus membrane perforation.

**Figure 4:**
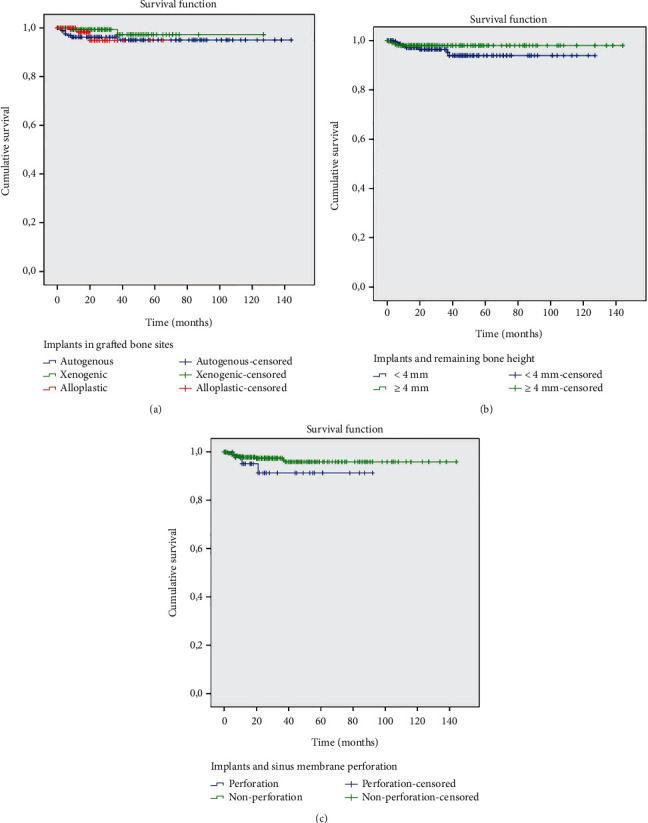
The Kaplan-Meier survival analysis. (a) Survival rate of the implants in grafted bone sites: autogenous, xenogenic, and alloplastic. (b) Relation between implant survival rates and remaining bone height: <4 mm and ≥4 mm. (c) Relation between implant survival rates and sinus membrane perforation.

**Figure 5:**
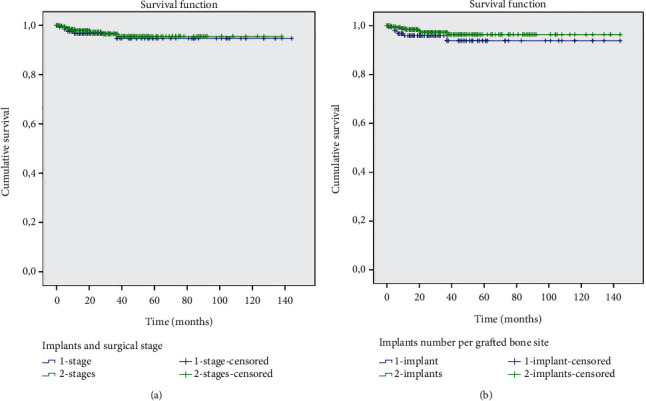
The Kaplan-Meier survival analysis. (a) Survival rate of the implants and type of surgical procedure: 1-stage and 2-stage. (b) Survival rate of the implants versus implant number per grafted bone site: 1 implant and 2 implants.

Figures 3–5 show the survival curves for the graft types and implants installed and indicate a cumulative survival rate above 90% for all combinations analyzed in this retrospective study.

## 4. Discussion

The maxillary sinus lift technique is employed for installation of implants in the posterior region of atrophic maxillae and achieves a favorable and predictable success rate [36, 51], indicating that this technique is able to overcome the challenges posed by bone resorption and pneumatization of the maxillary sinus [1, 2]. However, data from systematic reviews suggests some variability in success rates as a function of graft material used. One systematic review of maxillary sinus lift and implant success rates with follow-up ranging from 12 to 102 months showed a success rate of 92% for implants placed in autogenous grafts, 93.3% for allogenic bone, 81% for alloplastic material, and 95.6% for pure xenogenous bone [16]. In another two-year follow-up study, autogenous bone grafts were performed with simultaneous implant placement in 20 patients, resulting in an implant success rate of 100% [52].

To enable a better understanding of the medium- to long-term success rates of the maxillary sinus lift technique and the factors that may lead to failures, this 15 year retrospective study presents data from 757 implants in 472 bone graphs in a total sample of 384 patients. In our study, during a 15 year follow-up of 757 implants placed in grafts of maxillary sinuses, 7 implants were lost due to lack of osseointegration, and 14 were lost due to infection, resulting in an overall success rate of 97.2%. These results are consistent with those obtained in a six-year follow-up study by Lo Giudice et al. [34], 45 implants inserted in grafts had a success rate of 99.5%, and only one implant was lost before loading due to an acute graft infection after 24 days and two implants had no osseointegration and were removed after three months [34]. In terms of the success rate of the grafts, our study obtained a cumulative success rate of 98.3% (*n* = 472), with a mean follow-up of 3.6 years, in line with the results obtained in other studies [4, 6, 53–55]. These high success rates indicate that grafts provide a good structural basis to support dental implants and achieve a long-term success rate comparable to implants placed conventionally without graft procedures, regardless of the graft material used study, as suggested by Aghaloo and Moy [16].

Considering that autogenous, xenogenic, and alloplastic graft materials each have their own advantages and disadvantages, it is useful to consider the success rates as a function of graft material type. Grafting with autogenous bone is still considered the gold standard for maxillary sinus lift techniques because of its reliability, although these grafts can also be reabsorbed [19, 24, 52]. The success rate of the autogenous bone graft observed in our study was 97% across the 15-year period (*n* = 197), in line with the results obtained by Martuscelli et al. who achieved a 100% success rate in a small sample (*n* = 16) across a five-year period [54].

Despite these high success rates, there is a currently strong clinical tendency to avoid autogenous bone grafts in favor of organic or synthetic material [21, 22] due to the associated morbidity and postoperative complications at the potential donor sites [25, 56]. Among the available options for xenogenous grafts, bovine grafts are an attractive option because they are biocompatible, are osteoconductive, and have been used as a bone substitute for maxillary sinus lift with high success rates nine months after the graft [27]. However, biopsies of maxillary sinuses grafted with bovine xenogenous bone suggested that there may be a gradual diffusion of the biomaterial into the newly formed bone at the interface during resorption of the biomaterial [27]. Nonetheless, researchers observed that installation of the implants was still possible despite a mean contraction of 9.8% of the xenogenous bone graft volume eight months after the graft [55]. In our study, 99 out of 182 bovine bone grafts (45%) were suitable for implant installation, and the success rate was 99.5% after eight years of loading, which compares favorably with the success rate of 95.6% for pure xenogenous bone published in a recent meta-analysis [16].

The third option is grafting with alloplastic material, which is considered a safe, predictable, and noninvasive option based on histological and histomorphometrical comparison between alloplastic grafts and xenogenous grafts [26]. One study using hydroxyapatite as a bone substitute had a success rate of 100% for 25 implants placed into grafts after one to four years of function [23]. Although these results appear promising, other reports indicated that alloplastic materials only have osteoconductive potential, and thus, bone formation may require more time than with autogenous bone [25]. A comparative histological and histomorphometric analysis of alloplastic (biphasic calcium phosphate) and autogenous material by Tosta et al. [30] found that alloplastic material had lower tactile resistance than the autogenous bone over the newly formed bone. Thus, alloplastic material resulted in particles incorporated into the newly formed bone, whereas the autogenous bone showed bone neoformation patterns that were similar to the native area, although both graft materials allowed the installation of implants [30]. Another histological study showed remnants of alloplastic particles in close contact with the newly formed bone, several areas of resorption of the bone substitute, and remodeling of newly formed bone, which enabled the placement of implants [23]. Despite an initial 15% reduction of the alloplastic material in the first days after grafting, it subsequently maintained its volume during the first critical phase of bone remodeling (six months) and remained stable with high predictability, allowing placement of implants in alloplastic grafts [57]. In the results of our study, the alloplastic material (*n* = 92) was adequate for implant placement in 98.9% of the grafted sinuses.

In summary, the present study found that the xenogenous graft group presented the highest success rate (99.5%, *n* = 182), followed by the autogenous (97.0%, *n* = 197) and the alloplastic (98.9%, *n* = 92) groups; however, no statistically significant difference was found (*p* = 0.46). These results corroborate comparative studies that analyzed the performance of several bone substitutes and indicated that the different graft materials yield similar implant success rates [20, 30].

Some authors recommend that the sinus lift technique should be adapted as a function of the residual bone height, as follows: when the bone height is below 4 mm, two-stage surgical procedures should be carried out, and when the bone height is ≥4 mm, simultaneous installation of the implants is possible. A study that followed these recommendations had 100% success rate for 117 grafts in one-stage surgical procedures and 228 grafts in two-stage surgical procedures [51]. On the other hand, Felice et al. [32] carried out simultaneous graft and implant surgeries in the maxillary sinus with bone heights below 4 mm and reported a lower success rate (87.5%) in a small sample (*n* = 8). However, a multicenter randomized controlled trial by Bortoluzzi et al. [33] reported no significant differences between implants placed in 1- or 2-stage sinus lift procedures and obtained a 97.8% success rate for implants installed in bone sites with heights between 1 and 3 mm (*n* = 47). This is in line with the results obtained in our study, where 204 surgeries with simultaneous installment of implants were performed in patients with residual bone heights ≥ 4 mm, resulting in 6 implant losses, and 553 implants were installed in two surgical stages in patients with residual bone heights < 4 mm, resulting in with 14 implant losses. These results correspond to near-identical success rates of 97.0% and 97.4% for 1-stage and 2-stage sinus lift procedures, respectively. Therefore, we can conclude with a high degree of reliability that the combined effect of bone height and surgical technique did not influence the success rates in the present study.

The most common complication in maxillary sinus surgeries is the perforation of the sinus membrane [11]. However, when this complication is adequately corrected during the transoperatory phase, it does not compromise the success rates of the graft nor the implants [29, 37]. One study with 27 implants inserted in perforated sites achieved a 100% implant success rate despite this complication [39]. Conversely, a follow-up study with 12 cases with maxillary sinus perforation greater than 2 mm found that the success rate of the implants was significantly higher for sides where there was no perforation (100%) compared to the perforated sites (69.56%) [40]. Thus, even after correction, sinus membrane perforation may reduce the amount of available bone, decreasing the implant's success rate [40]. The membrane perforation rate in our study was 10.4%, and in all 49 cases, placements of grafts and implants was possible; the percentage of graft and implant loss in these regions was 2.04% (1 case) and 3.85% (3 cases), respectively, with no statistically significant difference. These results are more favorable than those reported in a recent systematic review, especially for the membrane perforation (30.6%) [38], and this can likely be attributed to the fact that perforations occurred during the surgical procedures were small (<10 mm) and were immediately repaired with a collagen-type membrane with adequate dimensions and biological properties.

This retrospective study describes the long-term results (up to 15 years) of two main clinical outcomes and is thus limited to providing a general view about the performance of the different graft types according to the (i) survival rate of bone graft materials over time, (ii) survival rate of the implants in grafted bone sites, and (iii) survival rate of the implants and type of surgical procedure: 1-stage and 2-stage. While the present study describes the long-term outcomes of numerous surgeries (472 grafts, 757 implants) performed in a single center with standardized surgical approaches involving a single brand of implants, no data was collected about the type, dimensions, and distribution in the posterior region of the implants and about the periodontal status of the jaws. In addition, another limitation of this retrospective study is the lack of assessment of bone stability parameters such as marginal bone level and endosinus bone gain. Future studies are needed to assess the influence of these parameters on the predictability of implants in the maxillary sinus grafted with different biomaterials.

## 5. Conclusion

Within the limitations of this retrospective study, the results obtained demonstrate that the maxillary sinus lift procedure is a viable and predictable alternative to enable implant installation, regardless of the residual bone height and the type of graft material used. The presence of sinus membrane perforation also did not significantly affect the success rates observed in this study.

## Figures and Tables

**Table 1:** Commercial brands of the bone grafts used in this study. HA: hydroxyapatite; TCP: tricalcium phosphate beta.

Bone graft material	Brand	Particle size	Dosage
Alloplastic (synthetic or inorganic material)	Alobone® Poros - Osseocon Biomateriais - Rio de Janeiro - Brazil	HA 250/1000 *μ*m	Vials: 5.0 g
	MBCP® Biomatlante - Vigneux-de-Bretagne – France	HA: 60%; TCP: 40% 1-2 mm and 2-3 mm	Vials: 2 cc or 30 cc
	BoneCeramic® Straumann - Headquarter - Basel -Switzerland	HA: 60%; TCP: 40% 0.5-1.0 mm	Vials: 1.0 cc-0.5 g or 1.9 cc-1.0 g
	Clonos® Neoortho - Curitiba - Paraná – Brazil	HA: 75%; TCP: 25% 0.5-1.0 mm (500-1000 *μ*m)	Syringes: 0.5 cc or 1.0 cc
	Neobone® Ceramed - Bella Vista – Santo Domingo - Rep. Dom.	HA: 75%; TCP: 25% 500-1000 *μ*m	Vials: 0.5 g and 1.0 g

Xenogenous (nonorganic bovine bone matrix)	Cerabone® Botiss Biomaterials GMBH – Zossen, Germany	HÁ 0.5–1000 mm	Vials: 05.ml or 1.0 ml
	Bio-Oss® Geistlich Pharma - Wolhusen, CH – Switzerland	Calcium: 35-40%; phosphate: 13.5-18.5% <5% moisture/organic material 1-2 mm	Vials: 0.5 g~1.5 cc; 1 g~3 cc; or 2 g~6 cc
	GenMix® Baumer - Mogi Mirim - São Paulo – Brazil	HÁ: 65-75% 25-35% moisture/organic material 0.25-1 mm	Vials: 0.75 cc, 1.5 cc, or 3 cc
	GenOx® Baumer - Mogi Mirim - São Paulo – Brazil	HA 0.50-1.0 mm	Vials: 0.5 cc = 0.5 g or 1.0 cc = 1.0 g

**Table 2:** Graft and implant success rates (%) and their association with (i) the type of graft material, (ii) height of residual bone, and (ii) membrane perforation (Chi-square test, *p* < 0.05).

Variables	Graft (success: yes/no, %)			*p* value	Implant (success: yes/no, %)			*p* value
	G1 (*n* = 197)	G2 (*n* = 182)	G3 (*n* = 93)		G1 (*n* = 312)	G2 (*n* = 298)	G3 (*n* = 147)	
Type of material								
	191/6 96.95%	181/1 99.45%	92/1 98.92%	*p* = 0.149	301/11 96.47%	293/5 98.32%	142/5 96.60%	*p* = 0.33
Height of residual bone								
<4 mm	85/4 95.51%	110/1 99.10%	54/1 98.18%	*p* = 0.238	85/3 96.59%	74/3 96.10%	37/1 97.37%	*p* = 0.94
≥4 mm	106/2 98.15%	71/0 100.00%	38/0 100.00%	^∗^	219/5 97.77%	218/5 97.76%	103/4 96.26%	*p* = 0.67
Membrane perforation (MP)								
Groups	15/1 97.96%	25/0 100%	8/0 100%	^∗^	21/1 95.45%	40/2 95.23%	14/0 100%	^∗^
Total	MP (*n* = 49)	No-MP (*n* = 423)			MP (*n* = 79)	No-MP (*n* = 678)		
	48/1 97.96%	416/7 98.35%		*p* = 0.842	76/3 96.20%	660/18 97.35%		*p* = 0.543

^∗^ *p*: test not applicable.

**Table 3:** Distribution of the implants according to the follow-up period.

Time between graft: clinical return (years)	*n*	%
≤ 3	191	50.5
3.1 to 5	97	25.7
5.1 to 8	59	15.6
>8	31	8.2
Total	378	100.0

## Data Availability

Data will be made available on request.
